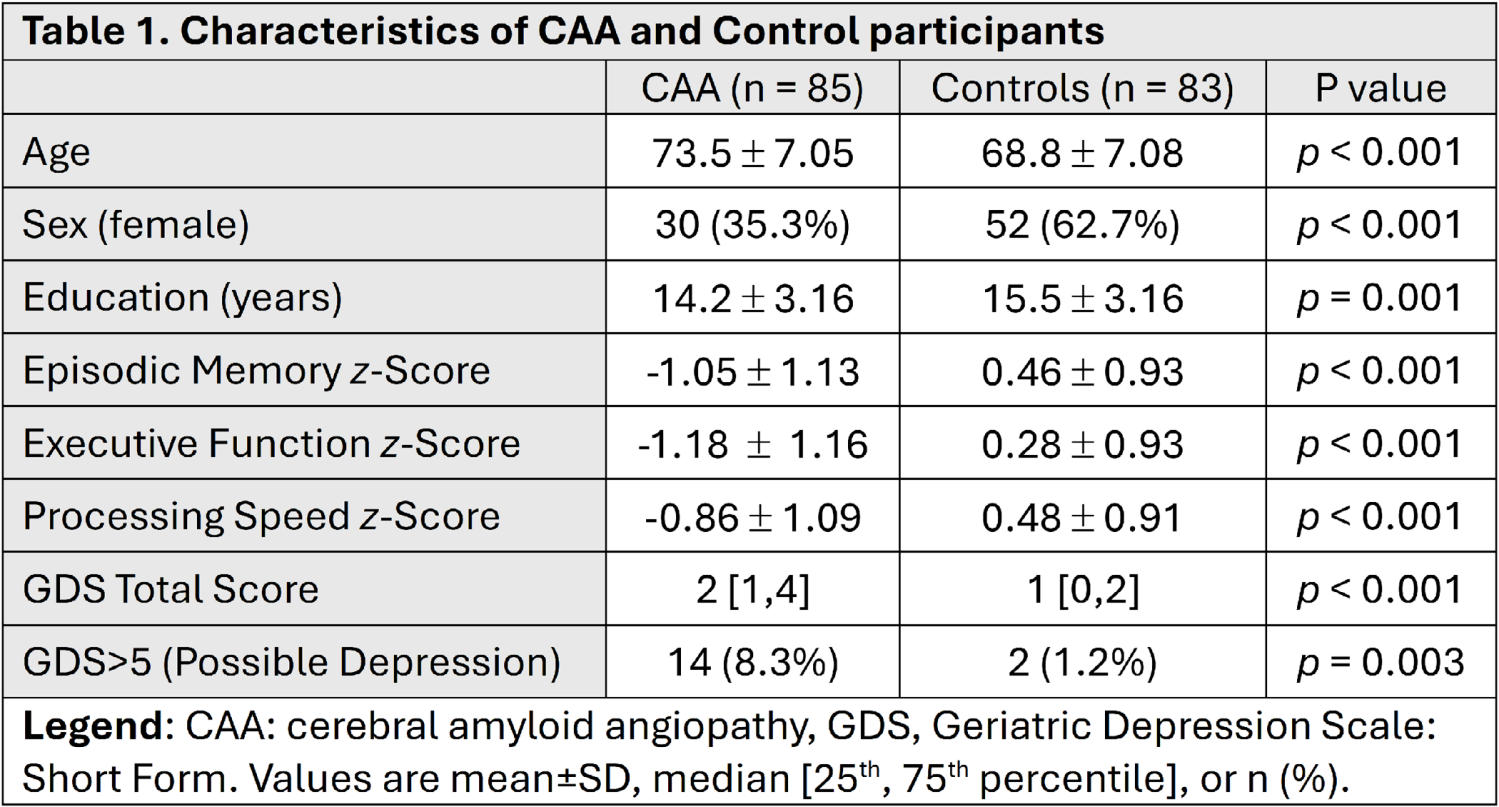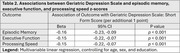# Associations Between Cerebral Amyloid Angiopathy, Cognitive Impairment, and Depressive Symptoms

**DOI:** 10.1002/alz70857_102973

**Published:** 2025-12-25

**Authors:** Nikita Nukala, Ryan T Muir, Andrew E. Beaudin, Cheryl R. McCreary, Myrlene Gee, Krista Nelles, Janina Valencia, Glen Jickling, Zahinoor Ismail, Richard Camicioli, Eric E. Smith

**Affiliations:** ^1^ University of Calgary, Calgary, AB, Canada; ^2^ Sunnybrook Research Institute, Toronto, ON, Canada; ^3^ University of Alberta, Edmonton, AB, Canada; ^4^ Hotchkiss Brain Institute, University of Calgary, Calgary, AB, Canada

## Abstract

**Background:**

Cerebral amyloid angiopathy (CAA) is linked to cognitive decline and dementia. Depression is associated with worse cognition, and depressive symptoms are among the most frequently reported neuropsychiatric symptoms in CAA. This study examined the individual associations between CAA, cognitive impairment, and depressive symptoms.

**Method:**

Cross‐sectional data from a prospective cohort study of participants with CAA and controls were analyzed. Neuropsychological testing was converted to *z*‐scores using test manual normative data for domains of episodic memory, executive function, and processing speed. The 15‐item Geriatric Depression Scale: Short Form (GDS) was used to identify participants with symptoms suggestive of depression, with GDS scores >5 categorized as “possible depression”. Multivariable linear or logistic regression was used to determine whether CAA group status and cognitive domain *z*‐scores were associated with possible depression, adjusting for age, sex, and education. Mediation analyses were used to determine the proportion of the effect of CAA on cognition that was mediated by possible depression, controlled for age, sex, and education.

**Result:**

There were 85 CAA and 83 control participants. The median GDS was 1 (interquartile range 0‐3) and 9.5% had possible depression (Table 1). Higher GDS score was associated with lower episodic memory, executive function and processing speed scores (Table 2). In multivariable logistic regression, CAA group status was associated with higher odds of having possible depression (OR 11.92, 95% CI 2.73 to 85.6, *p* = 0.003). Compared with controls, the CAA group exhibited poorer episodic memory, executive function, and mean processing speed performance (Table 1). However, the effect of CAA on cognition was mostly not mediated by possible depression: episodic memory, 5% (95% CI 0 to 18%, *p* = 0.06); executive function, 7% (95% CI 0 to 17%, *p* = 0.06); and processing speed, 2% (95% CI ‐5% to 10%, *p* = 0.58).

**Conclusion:**

Participants with CAA exhibited higher depressive symptoms and worse cognitive performance than controls. Patients with CAA should be screened and treated for depression. However, treating depression is unlikely to substantially improve cognitive function in CAA.